# A Non-Randomized Pilot Study on the Benefits of Baby Swimming on Motor Development

**DOI:** 10.3390/ijerph19159262

**Published:** 2022-07-28

**Authors:** Irene Leo, Silvia Leone, Raffaele Dicataldo, Chiara Vivenzio, Nada Cavallin, Chiara Taglioni, Maja Roch

**Affiliations:** 1 Department of Development and Socialization Psychology, University of Padua, 35131 Padua, Italy; leone.silvia91@gmail.com (S.L.); raffaele.dicataldo@unipd.it (R.D.); chiara.vivenzio@studenti.unipd.it (C.V.); maja.roch@unipd.it (M.R.); 2 Mamiù, Association for Baby-Swimming Activities, 35010 Padua, Italy; nadacavallin@gmail.com (N.C.); info.mamiu@gmail.com (C.T.)

**Keywords:** embodied cognition, motor development, baby swimming

## Abstract

According to the concept of “embodied cognition”, motor development should not be considered distant from cognitive and language processes. Motor development is essential in the first 1000 days of life, as the child explores and learns new information from the environment. Among motor activities, baby swimming allows infants to make movements that they are not able to perform on solid ground. Since movements become slower in water, the sensory perception of these movements is amplified. However, the relationship between early swimming experience and motor development has not yet been investigated. Therefore, we carried out a pilot study with the aim of exploring this relationship for the first time. To that end, 32 infants aged from 6 to 10 months were recruited. The Peabody Developmental Motor Scale-2 was used to assess motor abilities in healthy children who regularly carried out aquatic courses compared to children who never attended swimming practice. Independent-sample *t*-tests showed significant differences in favor of the group that performed infant swimming activities on measures of reflexes (*t* = −2.2, *p* < 0.05), grasping (*t* = −3.8, *p* < 0.001), fine-motor quotient (*t* = −3.4, *p* < 0.01) and total-motor quotient (*t* = −2.4, *p* < 0.05). Overall, in line with the embodied cognition perspective, these preliminary results are encouraging and allow us to investigate how motor development influences later language development.

## 1. Introduction

In recent decades, research in cognitive science has been revolutionized by the concept of embodiment, which places the body at the center of cognitive processes. Embodied cognition (EC) has become the core of a set of theories that emphasize the role of sensory and motor functions in cognition. Proponents of EC suggest that sensorimotor experience is critical for cognitive development [1]. Thus, there is a dynamic relationship between physical and mental experiences, suggesting an overlapping of cognitive and motor processes [2,3,4]. From this perspective, mental processes such as memory and perception overlap with action. The key to this interaction is to be found in the body, an agent influencing the nature of mental activity through contact with the environment. Among sensory experiences, action enables the necessary development of cognitive and intellectual skills [5]. Longitudinal studies support the embodiment thesis regarding cognition and highlight the benefits of early motor and sensory experiences in infants’ lives [6]. Accordingly, children experience high motivation to act if they receive enough and adequate stimuli. Perceptions, in turn, allow them to improve their actions. Thus, children can interact with their surrounding environment more effectively. Sensorimotor activity results in a basic integration of motor, tactile, and kinaesthetic sensations, as well as contact between the child’s body and the environment. Lagerspetz [7] and Zelazo [8] demonstrated the importance of exposing newborns to primordial types of physical exercise, circumscribed by maternal support, for the later development of motor skills. As a result, several researchers examined the impact of children’s mobility in the aquatic setting, which seems to encourage well-organized motor motion [9]. As a form of early motor experience, neonatal aquaticity provides a tactile and nonverbal connection between the mother and the baby, who is already familiar with the smell of her skin [10]. This practice includes touch therapy to provide children with maternal (or the closest caregiver) sensory stimulations. Touch therapy, combining massages, games, and early gymnastics, has been shown to help infants as young as 6–12 months old [11] and aged from 4 to 6 months [12]. However, the research on the advantages of aquatic environments for motor activity is scarce and contradictory. McGraw (1939) published cross-sectional findings indicating the occurrence of a disorganized motor phase in the first year of life (from 3 to 12 months of age) [9]. The author justified this slow learning by the occurrence of “cortical inhibition”. These early findings were subsequently refined and clarified by further investigations. In 1935, before this study, the author had already stated that “training with a single newborn at this period revealed quick learning” [13] (pp. 1–25), echoing contemporary research by Zelazo and Weiss, who emphasize the necessity of examining individual developmental trajectories in this vulnerable age range. After four months of training sessions, this disorganized behavior seemed to become rapidly organized; in particular, improvements in motor and cognitive performance occurred in children aged 16–20 months. An important and sudden transition in cognitive maturation occurs from the age of 12 months. This phenomenon would explain the difficulty encountered in detecting homogeneous abilities in such young children. As a result, the real and strong impact of aquaticity training may only be determined afterward.

The difficulty involved in assessing children at such a young age is mirrored by the more widespread choice of including older children in experimental designs, as in the study conducted by Nissim et al. (2014) [14], using children from 4 to 9 years of age. Numerous advantages can be derived from the use of such a larger sample (94 children), such as enabling a complete assessment of the developed abilities. In fact, this study showed how gross motor skills improved significantly in children who practiced aquatic activity compared to those who did not participate in any water motor exercise. The lack of available literature on infant swimming poses several challenges that prevent certain constraints from being addressed in the trial structure within the selected experimental designs. The limited dispersion of swimming courses and the acknowledged difficulties involved in gathering enough newborns—and furthermore of gathering those with the same beginning age—are likely to blame for the sometimes arbitrary age selection in this body of literature. This leads to a series of discordant or unexplainable results. This is the case for other findings supporting the effect of aquatic activity on motor development and coming from studies using children aged from 4 to 9 months on average. In this regard, Sigmundsson and Hopkins (2009) found reduced developmental risk in 4-year-old children who regularly attended infant swimming lessons at the age of 2–7 months [15]. In their study, Movement Assessment Battery for Children (MABC) scores revealed a better balance related to water activity. Finally, Dias et al. (2013) conducted a pilot study to investigate how baby swimming courses could affect early motor development in twelve children aged from 7 to 9 months [16]. The Alberta Infant Motor Scale (AIMS) scores in both the experimental and control groups showed a surprising improvement following a four-month aquatic treatment. These results still remain unsupported by any explanation. The authors claimed that group differences might lie in the limited sample size. These findings highlight the fact that having an age-homogeneous group is essential for this kind of study. The main drawback seems to be the difficulty in accounting for an extremely significant external variable: the number of months each sampled infant had actually practiced aquaticity. The children involved at the time of the evaluation could not have really participated in aquatic activities starting at the same age or for the same number of months. Therefore, in this investigation, we aimed to assess, for the first time, the advantages of aquaticity on infants’ motor development, despite the flaws in the preliminary sample gathering process. In particular, in this pilot study, we aimed to investigate motor development in children (ages 6 to 10 months) who regularly practiced neonatal aquaticity compared to those who never attended any baby swimming courses. This age range was chosen since there were local facilities available accepting swimming courses and ensuring entry for children older than six months. This may also help in providing a complete evaluation of the children’s fine and gross motor abilities, which is more challenging with younger subjects.

## 2. Materials and Methods

### 2.1. Participants

A total of thirty-two Caucasian children aged from 6 to 10 months (M = 8.5; SD = 0.92 months) were recruited for this pilot study. Among them, experimental subjects were selected from a group of infants attending neonatal aquaticity courses at the Mamiù Association (see below for details on aquaticity courses and schedules, http://www.mamiu.it/index.html) affiliated with the Department of Developmental Psychology and Socialization of the University of Padua. The inclusion criteria in the experimental group (Mean age = 8.1; SD = 1.1) included regular participation in aquatic motor activities once a week for 45 min. On the other hand, children assigned to the control group (Mean age = 8.9; SD = 0.34) never attended neonatal aquaticity courses or performed other motor activities. The Padua Registry Office (Italy) provided a list of potential children to be recruited for the control group and whose parents were contacted via postal letters. The selection of the control group revealed criteria of homogeneity with the experimental group. All the children come from the same urban area, social class (medium–high level), and from families with no history of autistic spectrum disorders, specific learning disorders, or specific language disorders. The selection of children from The Padua Registry Office for the control group followed the inclusion criteria concerning their age (a minimum of 6 months), the social class they belonged to, and their area of residence.

Results of the *t*-testing for independent samples showed a significant difference between the two groups in terms of age (*t* (2; 30) = 2.90; *p* = 0.007). Children in the experimental group were, on average, younger than their peers in the control group. After the study, twenty-eight children provided data and were included in the dataset for the analyses. Four children were excluded from the analysis as they had not completed certain PDMS-2 items due to irritation and crying. Both groups consisted of 14 children, with 9 males and 5 females in the experimental aquatics group and 8 males and 6 females in the control group. All the children were healthy, full-term children (38 to 41 weeks gestation) with no history of significant developmental events. Essential and necessary information about children was obtained from their parents, who filled out an ad hoc designed questionnaire and signed their consent before the start of the study, in accordance with the Declaration of Helsinki.

### 2.2. Assessment of Motor Ability

Children in the experimental and control groups were assessed at the Department of Developmental Psychology and Socialization of the University of Padua.

The Italian version of the Peabody Developmental Motor Scale-Second Edition (PDMS-2) [17] was administered to 32 children. This tool is an object-based performance test that provides for a multidimensional assessment of fine and gross motor abilities in pre-schoolers (from birth to 6 years). PDMS-2 is indeed useful in studying the effectiveness of various motor interventions, such as aquaticity in its early form of movement. The gross-motor quotient (QGM), the fine-motor quotient (QFM), and the total-motor quotient (QMT) are three global indicators of motor performance derived from this set of six subtests (249 items total). Children then performed age-specific motor activities related to reflexes, stationary position, locomotion, and object manipulation (for QGM), as well as grasping and visual-motor integration (for QFM). The QGM includes 151 items, whereas the QFM comprises 98 items. Items of the PDMS-2 are scored on a 3-point scale (0, 1, and 2). A score of 2 is assigned when the child performs the item according to the specified item criterion; a score of 1 indicates that the behavior is emerging but that the criterion for successful performance is not fully met; finally, a score of 0 indicates that the child cannot or will not attempt the item or that the attempt does not show that the skill is emerging. Therefore, the maximum raw scores of the subtests were different, ranging from 16 to 198. The developmental quotients for the GM, FM, and TM composites were then derived by summing the subtest standard scores and converting them to a quotient with a mean of 100 and a standard deviation of 15. The total-motor quotient, on the other hand, combined the preceding subtests’ findings and therefore represented the best estimate of global motor skills. The reliability coefficients for 3 composites and 6 subtests of the PDMS-2 (Cronbach α = 0.89–0.97, test-retest *r* = 0.82–0.93, and interscorer *r* = 0.96–0.99), reported in the test manual, showed that PDMS-2 is a reliable tool for the assessment of motor development.

## 3. Results

### Descriptive Statistics and Correlations among Variables

Descriptive statistics and listwise correlations among the standard scores for five PDMS-2 subtests and three global indices are reported in Table 1. Raw scores were converted into standardized scores through the conversion tables, as shown in the PDSM-2 manual. Performance levels in all the subtests were appropriate for age, covering a large range of scores, and none suffered from the ceiling effect. Children’s scores and the three global indices showed a good variability with standard deviations comparable to that of the national standardization sample. Therefore, we concluded that the range of performance was typical and unrestricted.

As can be seen from Table 1, scores for the PDMS-2 subtests showed moderate to strong correlations with the three global quotients (0.40 < *r* < 0.80), except for the area of visual-motor integration, which was not significantly correlated with the gross-motor quotient. Regarding the correlations between the five PDMS-2 subtests scores, the results showed that only stationary position scores were moderately correlated with locomotion and grasping, with *r* = 0.56 and *r* = 0.58, respectively. In order to investigate whether motor activity carried out in the aquatic context produced benefits in the motor development of children, we conducted a series of independent-sample *t*-tests, comparing the two groups (experimental vs. control) on each measure of motor development. As can be observed in Table 2, independent-sample *t*-tests show several significant differences in motor development between the experimental and control groups, showing that children involved in motor activity obtained better scores on measures of reflexes (*t* = −2.2, *p* < 0.05), grasping (*t* = −3.8, *p* < 0.001), the fine-motor quotient (*t* = −3.4, *p* < 0.01), and the total-motor quotient (*t* = −2.4, *p* < 0.05).

## 4. Discussion

This pilot study showed, for the first time, a potential link between infant motor development and neonatal aquaticity. The results indicated that children involved in water activities during the first year of life tended to have better motor skills. The most significant finding was the difference in scores between the experimental and control groups on the PDMS-2. Although all children achieved a satisfactory level of motor skills (at least a mean value of 8 in the five subtests and of 90 in the quotient scores), the regular aquatic practice seems to have facilitated the development of both fine motor skills (eye and hand coordination) and gross motor skills (reflexes). This investigation is thus the first step in understanding how early forms of motor activity could positively influence the acquisition of new skills in infants.

The EC viewpoint in the study of aquatic practice and motor development would explain these results from the perspective of environmental enrichment. The child’s internal perceptual and emotional experience involves interaction with an exterior world that is rich in stimuli. The parental figure is the starting point of these interactions. This becomes the focus of an emotional bond and trust in the child’s future attempts at exploration. The parent’s task of immersing the child in the outside world thus becomes a metaphor for infant aquaticity. When Rihatno et al. (2018) stated that the caregiver “plays an important role in motor development” [18] (pp. 391–395), they were referring to the aquatic environment and how it promoted the acquisition of motor skills in children. The caregiver enables the child to move towards a form of active behavior. The child thus initiates environmental exploration, following tactile, olfactory, visual, and auditory stimulation between their body and the mother’s body. In the first months after birth, the baby’s reflexes, and then their more coordinated motor actions, seem to be elicited by the amount and quality of the stimuli received.

The child’s development of new skills (quantitative development) and the level attained in each skill (qualitative development) are predictors of their physical and mental health [19,20]. Currently, experts are increasingly interested in understanding the benefits that aquatic therapies (baby spas in the form of touch therapy, massages, and hydrotherapy) have on physiological changes in infants. Indeed, studies to date indicate the importance of movement in water for typical development [12]. A study by Aditya (2014) revealed evidence supporting the relationship between aquatic environment and physical development [21]. The results of that investigation promoted the use of baby spas as a factor in improving weight gain and height in infants. Moreover, most of the correlations found in other studies were related to motor skill development and physiotherapy practices in baby spas [11,14,19,22]. Nevertheless, aquatics classes and baby spa treatments share a common ground, which could also explain the encouraging results of this pilot study. The aquatic environment is a multi-sensory stimulator, and its encounter with neuroplasticity, which is dense in the first months of a child’s life, could be responsible for the differences found in the motor domain between the experimental and control groups. Buoyancy, water density, and hydrostatic pressure [23] act as sensory stimuli on the vestibular and tactile systems [24]. Furthermore, the different depths to which each body is subjected in a pool allow individuals to experience an ever-changing gravitational terrain and to move in three-dimensional space [25]. By swimming in water in lower-gravity conditions, infants will finally gain control over their muscles [26].

Aquaticity is therefore of great importance, not only for general motor development but especially for the dimensions of fine motor skills and grasping. This result could provide the first step toward more general investigations in which motor development could be related to early language development. This is justified by several studies that focused on the relationship between motor development and language development [27,28,29,30,31]. According to Iverson (2009), motor acquisitions provide infants with an opportunity to practice skills relevant to language acquisition before they are needed for that purpose [32]. In infancy, there are significant changes in the ways in which the body moves in and interacts with the environment, and these may, in turn, affect the development of skills and experiences that play a role in the emergence of communication and language. Changes in motor skills (i.e., achievements and advances in posture, independent locomotion, and object manipulation) provide infants with a broader and more diverse set of opportunities for acting in the world. These opportunities provide contexts for acquiring, practicing, and refining skills that contribute, both directly and indirectly, to the development of communication and language. As stated before, the results indicated that children involved in water activities during the first year of life tended to show improved motor skills. If, as argued by Iverson (2009), changes in motor skills may produce changes in linguistic skills [32], it is possible to hypothesize that the benefits of water activities observed on motor skills may also extend to the language domain. The promising results obtained regarding stationary position and grasping are consistent with the results of previous research showing a relationship between fine motor skills and language. Independent sitting, walking, and grasping predicted later language development after the first year of age, in particular in relation to vocabulary size [27,29,33]. It is not yet clear to what extent and in what order the development of these motor dimensions contributes to language acquisition. However, fine motor skills contribute, together with perceptual and visual skills, to general cognitive development [31]. The use of the hands, both in the exploratory experience with objects and as a support for body positioning, certainly plays an important role in learning. EC promotes the notion of the harmonious development of children by which they continuously learn and experience contact with the outside world through their hands and through bodily movement.

The first year of life is a rather critical period for the child, who has the possibility to absorb many important inputs for physical and mental growth [34]. Neurodevelopmental research highlights the active participation of environmental stimuli in different domains, such as motor, language, and emotional development [35,36]. However, the open time window for dendritic arborization in infants is relatively brief, thus creating a major limitation in the assessment of their abilities. The age range used in this study (from 6 to 10 months of life) was chosen due to the difficulty involved in conducting a motor assessment battery, influenced by two major factors. Raising the minimum age criterion might guarantee the observation of more complicated movements than newborn reflexes, as the PDSM-2 contains age-specific motor tasks. This is compounded by the length of PDSM-2, which may cause stress in the youngest children. The exclusion of four children from the data analysis due to deep irritation contributed to the reduction in the sample. Having a wider range of ages in the future will allow us to refine our methodology and evaluate a baseline of ability with pretests. Other factors must be considered, given the difficulties observed in obtaining participants for aquatics training. The subjects’ ages are limited due to scheduling choices in the facilities: aquatics courses include children with a minimum age of 6 months. Furthermore, the facility offering the course is located in an upper-middle-class area, which excludes the possibility of recruiting a larger number of children (e.g., of the same age). Children’s parents would still have to be willing to make a financial contribution for the participation of their children in the course. In addition, the practice of aquatics has only recently been implemented in our national territory, and caregivers are sometimes not even aware of this opportunity.

Further studies adopting the “embodied cognition” perspective are needed in order to test this hypothesis since, to date, no studies have investigated the effects that water activities may have on both motor skills.

However, conducting non-randomized pilot studies involves risks associated with methodological choices. This type of study, which is becoming increasingly common [37], allows researchers to explore associations between variables and domains that are difficult to analyze. Although the feasibility of a pilot study is very often questioned, this type of investigation suits the refinement of the experimental design and structure that is appropriate to the sample and the research conditions. This first step in exploring the link between motor development and baby swimming could provide a starting point for those who wish to investigate cognitive development stages in infants. Longitudinal studies should be preferred in a context where, given the fast brain development of children, the effects of early motor programs on cognitive development need to be investigated. Despite the difficulties involved in practical management—given the costs of facilities and courses and the parents’ willingness to follow their children along this long path—the preparation of designs, including the pretesting of motor skills, is necessary. Preventive measures in terms of time management would allow for the assessment of younger children’s development (3–4 months of age) and their first reflexes up to linguistic tests around 3 years of age. The absence of a randomized trial seems to be a weakness of this study. The optimal evaluation conditions for upcoming experimental designs would be based on the selection of a more homogeneous sample in terms of age to be assessed at least at two different time points: a first pre-baseline assessment at T_0_ (with all the infants having the same month of age who have not yet begun practicing aquatics) and the following test at T_1_ (after a few months of practice), followed by follow-up sessions for longitudinal studies. In this way, the observation of motor skills, language, and cognitive development would provide insights into the potential effectiveness of aquatics in children aged 0–36 months. Nevertheless, the assessment of the children’s baseline levels, referring to their motor skills, was carried out, in this study, based on the parents’ reports. Infants’ motor abilities were found to be in line with their age, given the absence of any particular movement dysfunction. Furthermore, at an early age, the control of external environmental variables should not be underestimated. In this case, ad hoc questionnaires would investigate in more detail how and whether the caregivers’ life habits and behavior could affect the children.

Finally, of no less importance are the baby’s habits. The ad hoc questionnaires used in this study certainly investigated birth-related factors, such as the week of gestation, the type of birth, and the baby’s weight. No differences were found between children with regard to their period of birth (no pre-term births), nor did we find any substantial differences in weight. As mentioned above, the behavioral analysis performed at such an early age is challenging, as external variables limit the control over the assessment of children. Some of these interfering factors, to be evaluated in the future, would be related to the children’s daily motor habits, such as sitting or crawling time and time spent with caregivers or nursery figures.

## 5. Conclusions

In conclusion, the results of this pilot study provide further evidence on the potential influence of early aquatic activities on the motor development of children, showing that infants in the experimental group, although younger than children in the control group, achieved greater levels of motor development after participating in water activities than those reached by the control group. Although these findings are not definitive and only represent part of an initial exploratory investigation, they seem to support the notion, consistent with the findings of previous studies (despite our methodological limitations), that aquatics could influence early motor development in infants and toddlers. This study may thus be useful in filling the gap in the literature on aquaticity, but above all on infants’ ability to embark on healthy developmental pathways, with the caregiver–child interaction playing a role that should not be underestimated. Future investigations could shed light on the link, throughout the first year of life, between motor, language, and cognitive development promoted by aquaticity.

## Figures and Tables

**Table 1:** Descriptive statistics and correlations among variables.

	2.	3.	4.	5.	6.	7.	8.	M (SD)	Range
1. Reflexes	0.18	0.08	0.38	0.23	0.61 **	0.42 **	0.60 **	9.5 (1.6)	6–13
2. Stationary position		0.56 **	0.58 **	0.12	0.76 **	0.47 *	0.66 **	11.3 (1.2)	9–13
3. Locomotion			0.33	0.26	0.74 **	0.40 *	0.59 **	10.3 (1.5)	7–14
4. Grasping				0.19	0.55 **	0.73 **	0.60 **	10.9 (1.5)	6–13
5. Visual-motor integration					0.21	0.80 **	0.48 **	9.7 (1.8)	7–13
6. QGM						0.51	0.85 **	103 (6.9)	89–114
7. QFM							0.73 **	100 (6.7)	88–112
8. QMT							1	101 (7.4)	85–115

Note. * *p* < 0.05; ** *p* < 0.01.

**Table 2:** Group comparison of Peabody Developmental Motor Scale Second Edition (PDMS-2) results.

Variables	Experimental Group (*N* = 14)		Control Group (*N* = 14)		*t*-Test Independent Sample	
	Range	Mean (SD)	Range	Mean (SD)	*t* (*df*)	*p*
1. Reflexes	8–13	10.2 (*1.6*)	6–11	8.8 (*1.4*)	−2.2 (*24*)	0.03
2.Stationary position	10–13	11.6 (*0.93*)	9–13	11 (*1.4*)	−1.3 (*25*)	0.18
3. Locomotion	9–14	10.3 (*1.5*)	7–13	10.3 (*1.6*)	−0.08 (*25*)	0.93
4. Grasping	10–13	11.8 (*0.86*)	6–12	10 (*1.5*)	−3.8 (*26*)	0.001
5.Visual-motor integration	7–13	10.2 (*1.6*)	6–13	9.2 (*1.9*)	−1.5 (*26*)	0.14
6. QGM	93–114	105 (*6.1*)	89–114	102 (*7.5*)	−1.2 (*24*)	0.23
7. QFM	94–113	104.5 (*5*)	88–107	97 (*6.2*)	−3.4 (*26*)	0.002
8. QMT	96–115	105 (*5.2*)	85–112	98.4 (*8.2*)	−2.4 (*24*)	0.02

Note: QGM = gross-motor quotient; QFM = fine-motor quotient; QMT = total-motor quotient.

## Data Availability

The data that support the findings of this study are available from corresponding author, I.L., upon reasonable request.
